# Supplementary material for: Determination of the Influence of Multiple Closed Recycling Loops on the Property Profile of Different Polyolefins
Source: Polymers (Basel). 2022 Jun 15;14(12):2429. doi: 10.3390/polym14122429 (PMC9231274; doi:10.3390/polym14122429)
Supplement: Supplementary file 1 [file polymers-14-02429-s001.zip › polymers-1740235-supplementary.pdf]

polymers

MDPI

Supplementary Material

Determination of the influence of multiple closed recycling loops on the property profile of different polyolefins

Johanna Langwieser[1, 2]*, Andrea Schweighuber[3], Alexander Felgel-Farnholz[3], Christian Marschik[1], Wolfgang Buchberger[3] and Joerg Fischer[2]

[1] Competence Center CHASE GmbH, Altenberger Straße 69, 4040 Linz, Austria
[2] Institute of Polymeric Materials and Testing, Johannes Kepler University Linz, Altenberger Straße 69, 4040 Linz, Austria
[3] Institute of Analytical and General Chemistry, Johannes Kepler University Linz, Altenberger Straße 69, 4040 Linz, Austria
* Correspondence: johanna.langwieser@chasecenter.at; Tel.: +43 664 9693520

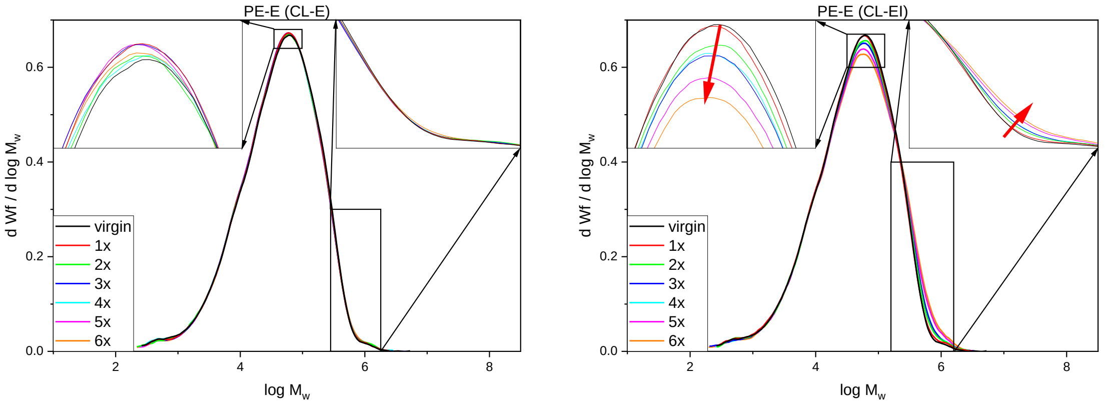

(**a**) (**b**)

Figure S1. MMD of PE-E: (a) MMD after each loop of the CL-E and (b) MMD after each loop of the CL-EI.

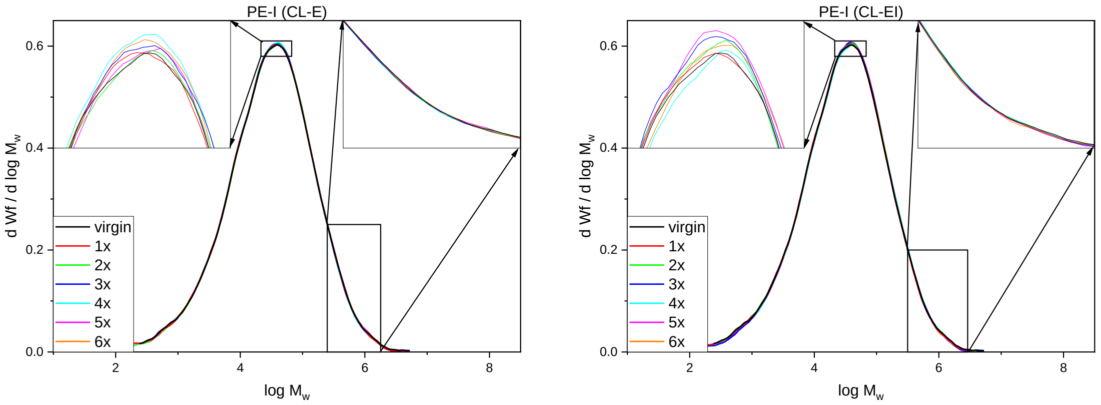

(**a**) (**b**)

Figure S2. MMD of PE-I: (a) MMD after each loop of the CL-E and (b) MMD after each loop of the CL-EI.

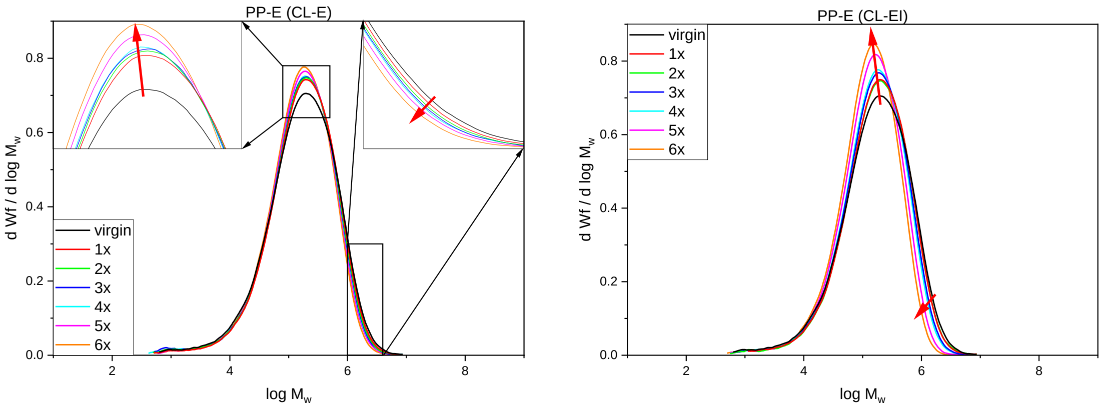

(**a**) (**b**)

Figure S3. MMD of PP-E: (a) MMD after each loop of the CL-E and (b) MMD after each loop of the CL-EI.

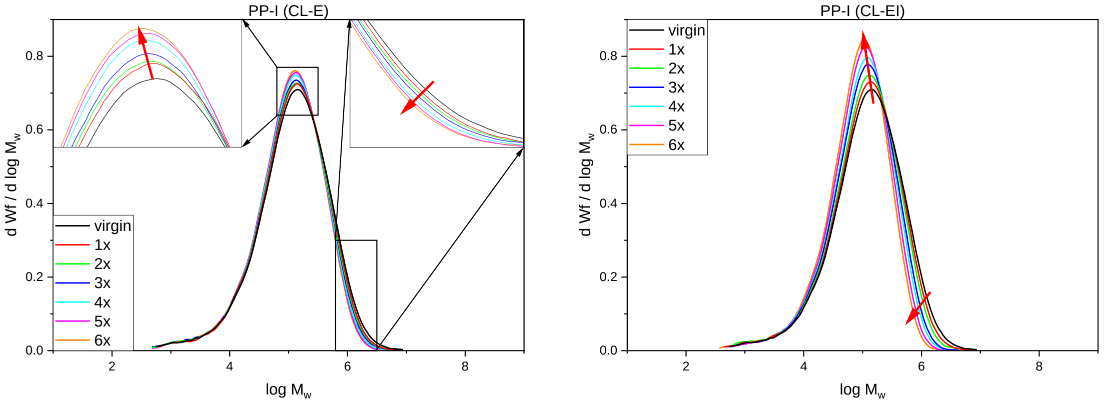

(**a**) (**b**)

Figure S4. MMD of PP-I: (a) MMD after each loop of the CL-E and (b) MMD after each loop of the CL-EI.

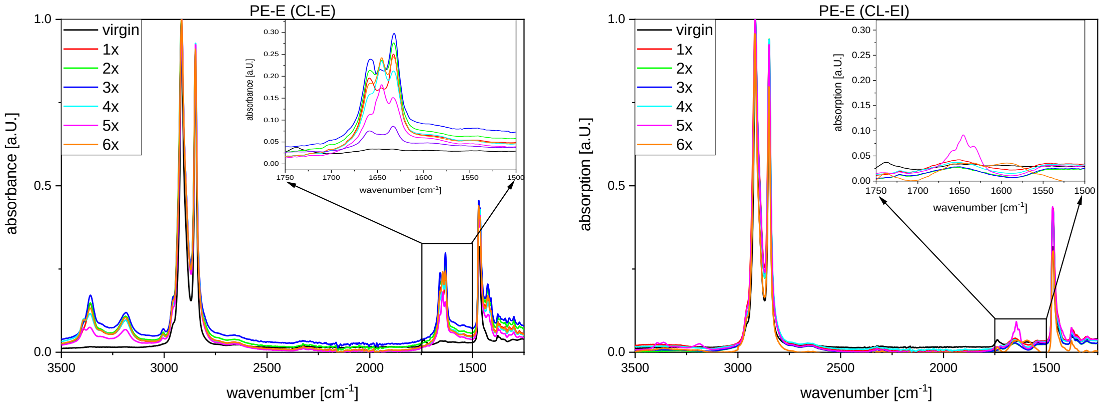

(**a**) (**b**)

Figure S5. PE-E: IR absorbance spectra and enlarged spectra in the wavenumber range from 1750 to 1500 cm^{-1} of the (a) CL-E and (b) CL-EI

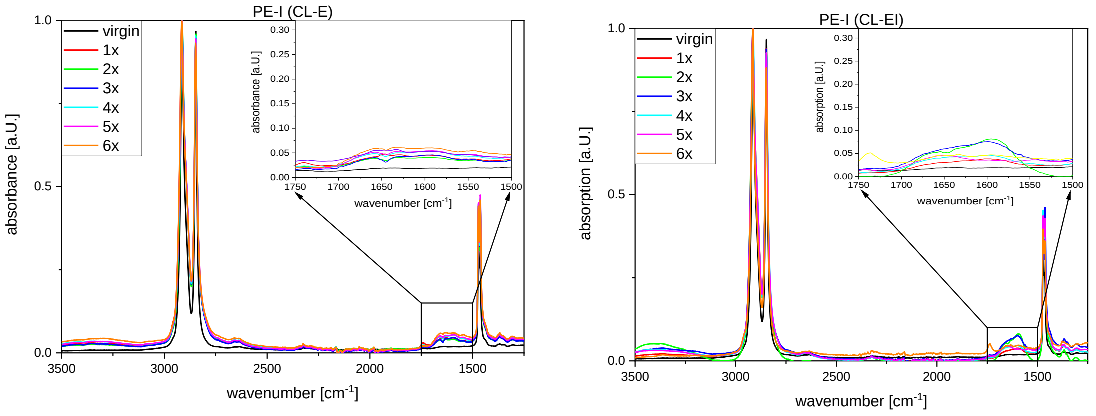

(a) (b)

Figure S6. PE-I: IR absorbance spectra and enlarged spectra in the wavenumber range from 1750 to 1500 cm^{-1} of the (a) CL-E and (b) CL-EI

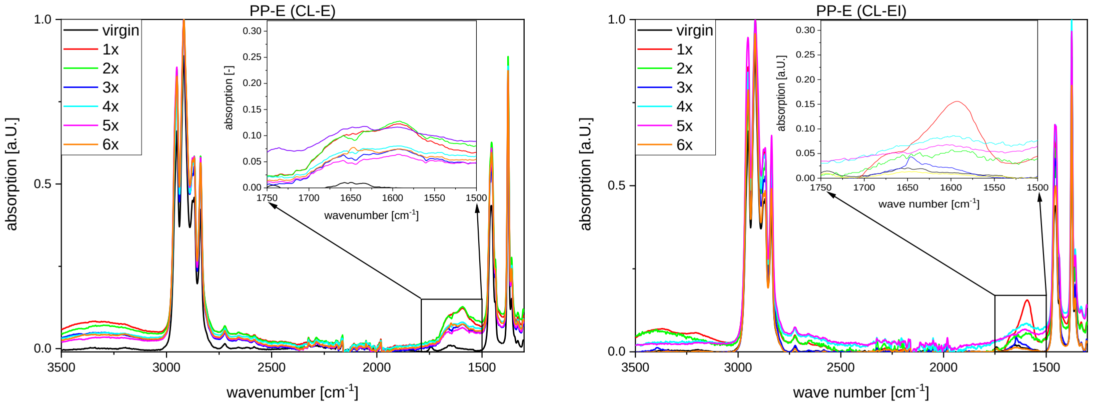

(a) (b)

Figure S7. PP-E: IR absorbance spectra and enlarged spectra in the wavenumber range from 1750 to 1500 cm^{-1} of the (a) CL-E and (b) CL-EI

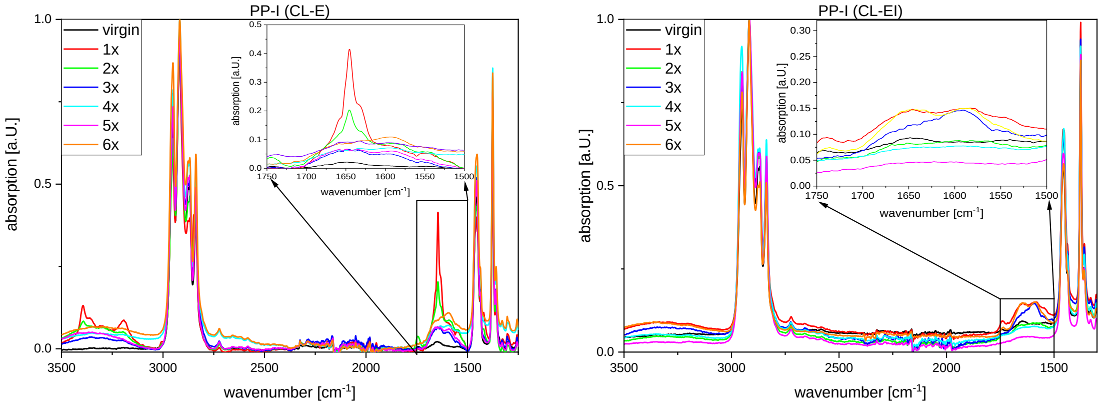

(a) (b)

Figure S8. PP-I: IR absorbance spectra and enlarged spectra in the wavenumber range from 1750 to 1500 cm^{-1} of the (a) CL-E and (b) CL-EI

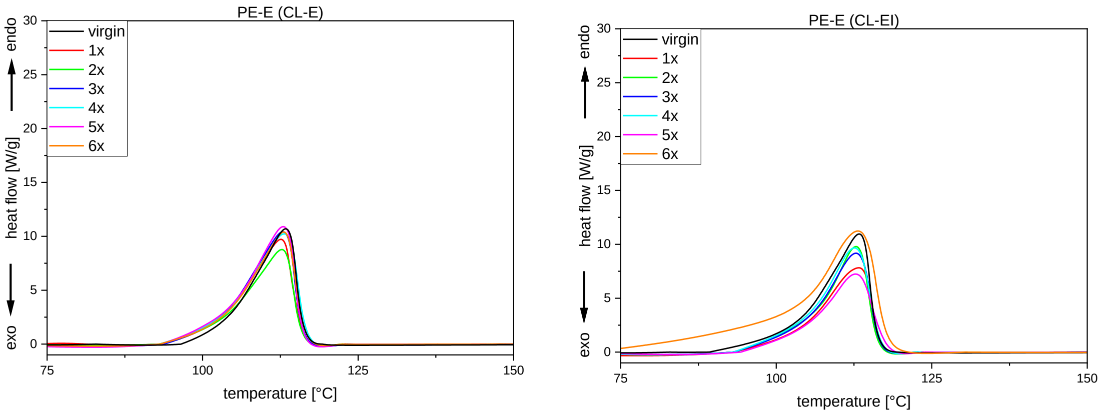

(**a**) (**b**)

Figure S9. Melting peaks of the PE-E after each loop for (a) CL-E and (b) CL-EI.

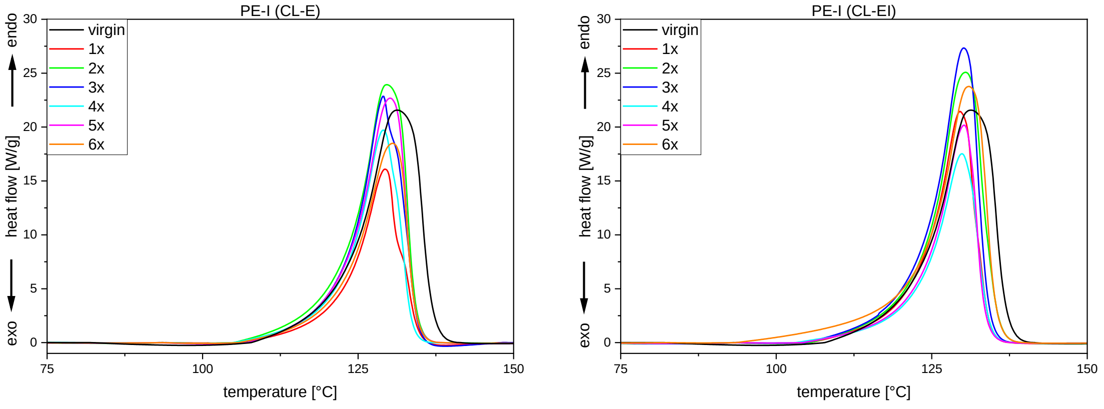

(**a**) (**b**)

Figure S10. Melting peaks of the PE-I after each loop for (a) CL-E and (b) CL-EI.

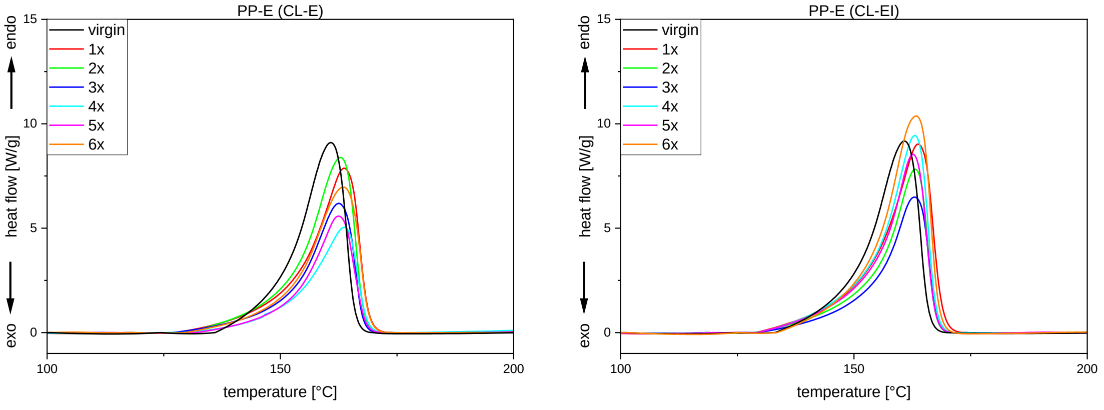

(**a**) (**b**)

Figure S11. Melting peaks of the PP-E after each loop for (a) CL-E and (b) CL-EI.

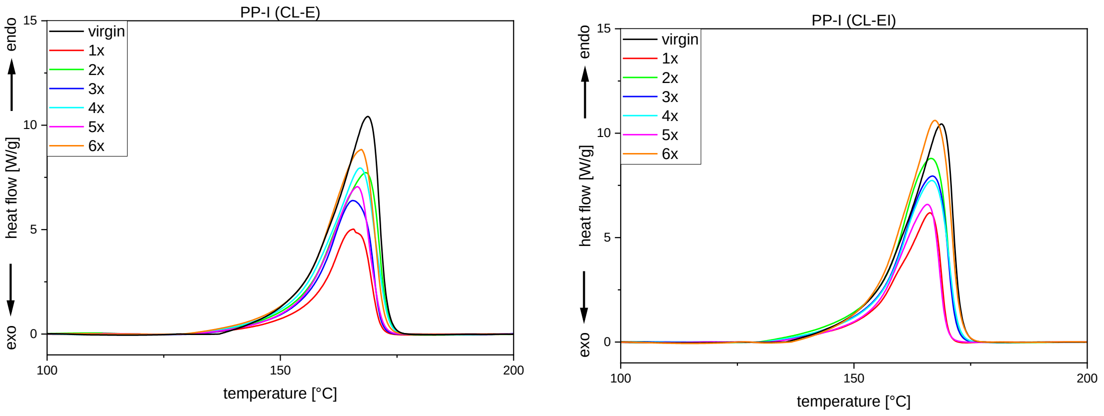

(**a**) (**b**)

Figure S12. Melting peaks of the PP-I after each loop for (a) CL-E and (b) CL-EI.

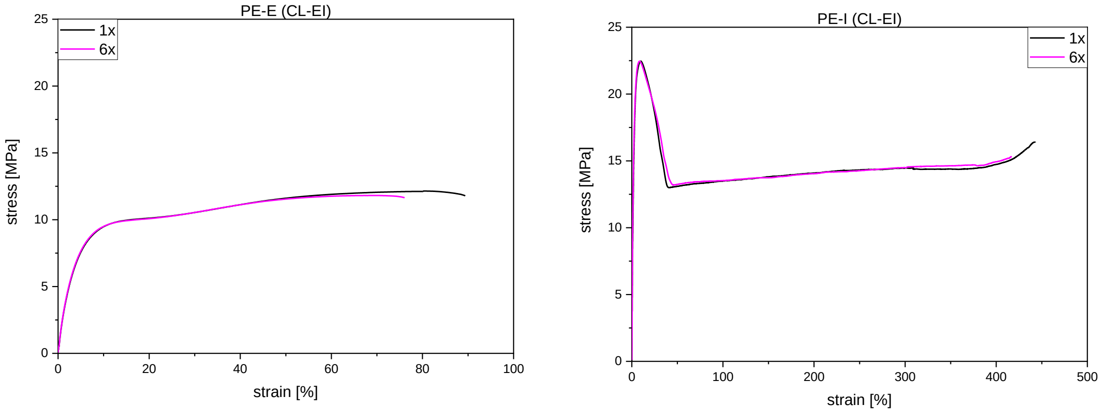

(**a**) (**b**)

Figure S13. Melting peaks of the PP-I after each loop for (a) CL-E and (b) CL-EI.

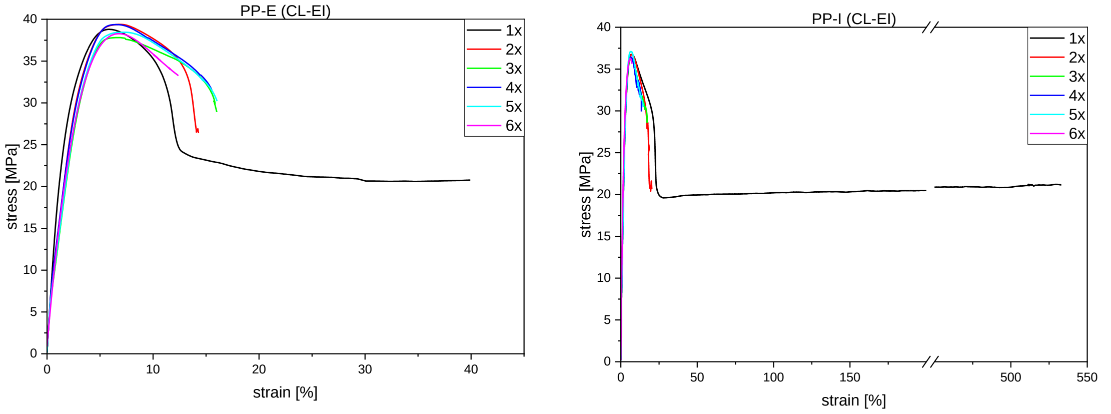

(**a**) (**b**)

Figure S14. Melting peaks of the PP-I after each loop for (a) CL-E and (b) CL-EI.
